# Telomere fusions associate with coding sequence and copy number alterations in CLL

**DOI:** 10.1038/s41375-019-0423-y

**Published:** 2019-02-22

**Authors:** Laura Escudero, Kez Cleal, Kevin Ashelford, Chris Fegan, Chris Pepper, Kate Liddiard, Duncan M. Baird

**Affiliations:** 1 Division of Cancer and Genetics, School of Medicine, Cardiff University, Cardiff, UK; 2 Brighton and Sussex Medical School, Sussex University, Brighton, UK

**Keywords:** Chronic lymphocytic leukaemia, Cancer genomics

## To the Editor:

Short-dysfunctional telomeres are detected prior to clinical progression in chronic lymphocytic leukaemia (CLL) and result in chromosomal fusions that propagate genome instability, driving disease progression. To investigate the impact of telomere dysfunction on the CLL genome, we performed a large-scale molecular characterisation of telomere fusion events in CLL B-cells. A cohort of 276 CLL patient samples was selected for analysis based on short telomere length (TL) profiles, with the majority (97%, *n* = 269) having mean TL within the previously-defined fusogenic range in CLL [1]. Patient samples were screened for the presence of telomere fusions using a single-molecule telomere fusion assay [2] modified to include the 5p telomere (Supplementary Figure 1). Telomere fusions were detected in 72% (198/276) of the samples, which were subsequently arbitrarily stratified by fusion frequency (Supplementary Table 1). Fusions were detected for all telomeres assayed, including the 5p telomere, for which fusions were present in 23% (40/177) of patient samples (Supplementary Figure 2, Supplementary Table 2).

**Fig. 1:**
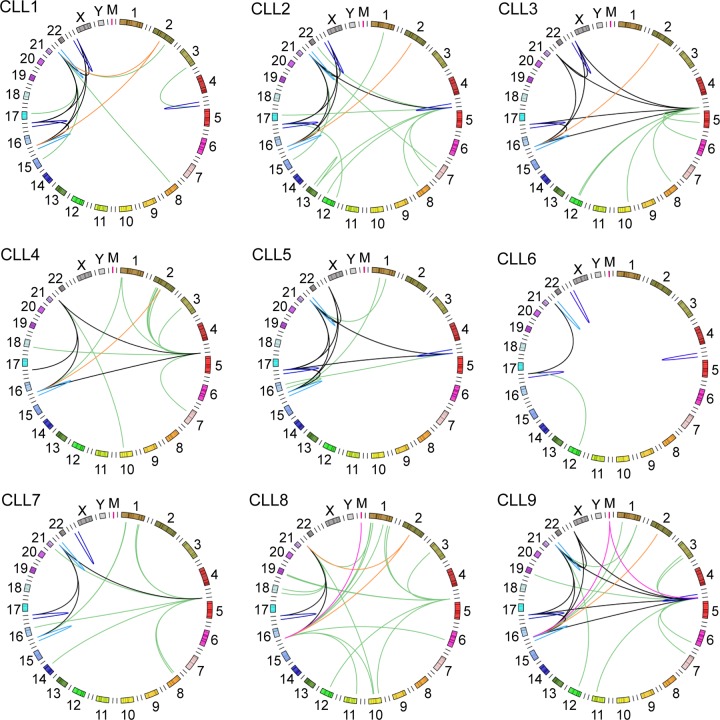
Signature of telomere fusions for 9 CLL patient samples. Circos plots showing the validated results obtained from the inter-chromosomal and intra-chromosomal telomere fusion analysis from nine CLL patient samples. Circos plot with each chromosome and its telomeres (1p telomere, Chr1, 1q telomere) around the circle orientated clockwise. Additional notches indicate linkages specifically aligning with subtelomeric sequence references derived from Stong et al. [12]. Colour code: telomere-telomere inter-chromosomal (black), telomere-telomere intra-chromosomal for 5p, 17p and XpYp (blue), inter-chromosomal or intra-chromosomal for 16p and 21q families (light blue), and inter-chromosomal telomere-genomic (green), telomere-2q13 (orange) and telomere-ChrM (pink). Telomere fusion events with unknown sub-telomeric sequence were not included

High-resolution characterisation of single-molecule amplified telomere fusions from nine CLL patients with the highest fusion frequency was performed by Illumina HiSeq4000 paired-end sequencing. Following a customised bioinformatics analysis pipeline [3] and manual curation (Supplementary Figure 3), 914 unique telomere fusions were resolved (Supplementary Figure 4), of which 19% (172/914) involved the 5p telomere (Supplementary Figure 5). Intra- (sister-chromatid) or inter-chromosomal telomere fusion events were identified, as well as recombinations involving non-telomeric loci incorporated into telomere fusions (Fig. 1; Supplementary Figure 4, Supplementary Table 3). These captured loci included; the ancestral telomere at Chr2q13-14 (*n* = 11), mitochondrial DNA (*n* = 4) and other non-telomeric genomic loci (*n* = 78). Complex inter-chromosomal events involving multiple disparate loci were also detected (*n* = 7) (Supplementary Figures 5–9).

Distinct signatures of telomere fusions across the genome could be described for each CLL patient sample (Fig. 1). Two patients (CLL3 and CLL6) displayed simple signatures, defined by the presence solely of intra-chromosomal and/or inter-chromosomal telomere-telomere fusions. In contrast, the CLL8 sample telomere fusion profile revealed abundant genomic linkages, including with the ancestral telomere at 2q13 and mitochondrial DNA. Samples CLL1, CLL2, CLL4, CLL5, CLL7 and CLL9 were characterised by complex signatures with a combination of most or all categories of telomere fusion events identified in this study (Fig. 1; Supplementary Table 4).

Telomere dysfunction is associated with increased genomic instability and disease progression in CLL [1, 4], therefore a comprehensive analysis of all patient-derived telomere fusions with non-telomeric genomic loci was undertaken. Locations and junction sequences pertaining to all 93 (10% total fusions) identified inter-chromosomal fusions were investigated to determine commonality of global or local sequence context as well as providing evidence for the engagement of specific DNA repair processes. These inter-chromosomal genomic fusions were less abundant than pure telomeric inter-chromosomal fusions that represented 38% of all fusions characterised.

**Fig. 2:**
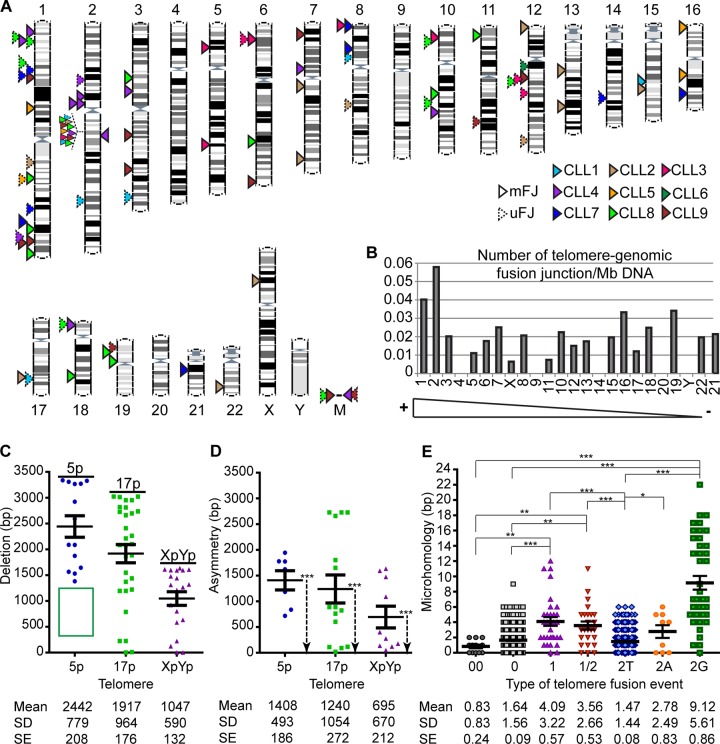
Characterisation of telomere fusions detected across the genome. **a** Validated inter-chromosomal telomere fusion events (*n* = 93) on a karyotype map generated in Ensembl. Telomere fusions with genomic, ancestral telomere 2q13 and mitochondria DNA/Chr. Each colour represents a different patient sample. Continuous arrow-heads indicate mapped fusion junctions (mFJ) and discontinuous arrow-heads represent unmapped fusion junctions (uFJ, location of the read represented). **b** Number of validated inter-chromosomal telomere-genomic fusion junctions per Mb of DNA for each chromosome ordered by length (size obtained from Ensembl). **c** Sister-chromatid deletion and **d** asymmetry for the 5p, 17p and Xp chromosome ends of intra-chromosomal fusion events. Green box highlights the CpG island on the 5p sub-telomere. Location of the fusion primer indicated, determines the limit of the assay from the telomere. **d** Level of asymmetry was determined by calculating the deletion difference between each chromatid of the same fusion event. **e** Microhomology (bp) at the fusion junction was compared for the distinct type of events: TTAGGG-CCCTAA (00), Sub-telomere-TTAGGG (0), intra-chromosomal (1), intra-chromosoma or inter-chromosomal of 16p-16p and 21q-21q families (1/2), inter-chromosomal telomeric fusion events (2T), inter-chromosomal fusions with the ancestral telomere at 2q13 (2A) and inter-chromosomal fusions with genomic loci (2G). Mean, SD and SE are indicated below

Inter-chromosomal fusions with non-telomeric genomic loci were identified in all nine CLL patient samples. Individual events were validated by manual sequence analysis, revealing 68% (63/93) had fusion junctions covered by junction-spanning sequence read pairs (mFJ) and 32% (30/93) had unmapped junctions (uFJ). Each fusion junction location was depicted on the ideogram in Fig. 2a. Notably, the loci disrupted by telomere fusions (summarised in Supplementary Table 5) were not randomly distributed throughout the genome since there was no simple correlation with chromosome length (*r*^2^ = 0.44) or coding gene density of the respective chromosomes (*r*^2^ = 0.32) (Fig. 2b, Supplementary Figure 10). However, loci with previously-reported copy number aberrations in CLL [5] were found to be incorporated into telomere fusions, including 2p15, 2p11.2 (2 events), 2q13 (11 events), 6q22.31, 11q22.2 and 18q21.32 (single events). In addition, a complex telomere fusion was detected involving four distinct loci including 13q14.2 that is frequently deleted in CLL (Supplementary Figure 4B).

Inter-chromosomal telomere fusions occurred within coding DNA more frequently than expected by chance. Over half (57%) of mFJ were within introns and exons of protein-coding genes (Supplementary Table 5), significantly higher (Chi-squared analysis *p* = 0.0024) than the average 42% gene content of the human genome (based on the hg19 RefGene). We also observed 15% and 9% mFJ fused with Common Fragile Sites (CFSs) and Alu elements, respectively; however, these were similar to the proportion of CFSs (15%) and Alu sequences (11%) identified across the human genome [6].

All 31 protein-coding genes disrupted by telomere fusions with mFJ were further investigated for potential association with CLL pathogenesis (Supplementary Table 6). An enrichment in genes overexpressed in CD38^+^ patient CLL B-cells was revealed using GSEA Gene Set Enrichment Analysis (GSEA, v5.2) Molecular Signatures Database (MSigDB) [7]. This gene set included *HTR7*, *KIF26B* and *LPHN1* (*p*-value 1.5e^−6^; FDR *q*-value 2.7e^−2^) —genes previously found to be upregulated in CD5^+^/CD19^+^/CD38^+^ CLL cells associated with worse patient prognosis, compared with patient-matched CD5^−^/CD19^−^/CD38^−^ CLL cells in a panel of six patient samples [8]. Strikingly, 36% (11/31) of all genes disrupted by a telomere fusion event for which the junction could be validated were classified as expressed or associated with B lymphocytes or CLL B-cells. These genes included *CD8A*, *RORA*, *TESPA1*, *DMD*, *NOX5*, *NTF3*, *EVI5* and *FTO* (Supplementary Table 7) with documented pathological relevance. A significant enrichment in genes possessing binding motifs matching the B-cell-expressed homeobox transcription factor, HNF1α (TCF1) [9], within their promoters was also identified (*DMD, RORA, NTF3* and *HTR7*; *p*-value 2.51e^−5^; FDR *q*-value 1.31e^−2^; Supplementary Table 8). Furthermore, a noteworthy association of fusion-disrupted genes with gene sets over-expressed in other types of cancer including breast and liver was also revealed by these analyses.

We have previously shown that intra-chromosomal telomere fusion is accompanied by extensive resection that results in asymmetric deletion of the participating sister-chromatids [3]. To assess whether this was true for CLL B-cells, the extent of DNA end-processing at each sister-chromatid was examined for intra-chromosomal fusions with mFJ. The distance from the start of the telomere repeat sequences to the fusion junction for each of the chromatids involved in the fusion event was determined and the difference calculated to obtain a measure of asymmetry (Fig. 2c, d; Supplementary Tables 9-10). The uneven distribution of fusion junctions across the 5p sub-telomere (*n* = 14) is consistent with the location of a CpG island and suggests that the GC-rich sequence may hamper the detection of 5p fusion events (Supplementary Figure 11). Thus, 5p telomere fusions may be under-represented in the data and may have an even greater impact on CLL disease than presently recognised. In contrast, telomere fusion junctions were effectively captured across the 17p (*n* = 30) and XpYp (*n* = 20) telomeres (Fig. 2c). Asymmetry of sister-chromatids was observed for 5p, 17p and XpYp with a mean of 1408 bp, 1240 bp and 695 bp, respectively (Fig. 2d). The degree of asymmetry was significantly greater than the theoretical value 0 (one sample *t*-test, *p* < 0.001). This indicates that fusion occurs between sister-chromatids of different lengths in CLL B-cells, consistent with our observations in other models [2, 3, 10]. No significant differences were found in the extent of asymmetry between the 5p, 17p and XpYp chromosome ends (Kruskal-Wallis, *p* = 0.1661).

High-resolution analysis of each CLL mFJ was performed to investigate candidate DNA repair mechanisms that may underlie distinct types of telomere fusion events. Insertions of templated, untemplated and/or potential telomere variant repeat sequences were observed at 6% (50/796) of mFJ: 23/50 for Telomere-Sub-telomere, 4/50 for intra-chromosomal, 2/50 for intra/inter, 19/50 for telomeric inter-chromosomal, 1/50 for telomere-Chr2q13 and 1/50 for telomere-ChrM fusions. Insertions ranged from 1-21 nucleotides with a mean of 4.5 nucleotides. In contrast, no insertions were identified at fusions with non-telomeric loci. Statistically-significant differences in the extent of microhomology usage at fusion junctions were determined for the different types of telomere fusion events (Kruskal-Wallis *p* < 0.001 and Dunn’s Multiple Comparison Test) (Fig. 2e; Supplementary Table 11). Inter-chromosomal fusions with non-telomeric loci (mean = 9.1 bp; *n* = 43), together with intra-chromosomal sister-chromatid events (mean = 4.1 bp; *n* = 32), displayed the greatest amounts of junction microhomology. In contrast, very low or an absence of microhomology at the fusion point was observed for inter-chromosomal telomeric fusions (mean = 1.5 bp; *n* = 315), Telomere-Telomere (TTAGGG-CCCTAA; mean = 0.8 bp; *n* = 12) and Telomere-Sub-telomere (mean = 1.6 bp; *n* = 303) subgroups. Long tracts of microhomology of up to 23 bp, were observed at inter-chromosomal fusion junctions with non-telomeric loci (Fig. 2e). When the usage of microhomology was >10 bp, the sequence was enriched for the repeat unit of (AC)_n_ (Supplementary Figure 5); 40% (6/15) of events that contained at least (AC)_5_ (motif ACACACACAC), consistent with repair utilizing single-stranded annealing [11].

Taken together, our data reveal the impact of short-dysfunctional telomeres on the evolving CLL genome, generating tumour heterogeneity that may affect patient prognosis. We have revealed that dysfunctional telomeres predominantly fuse with protein-coding DNA including genes expressed in CLL B-cells and other tumours. We have also identified complex telomere fusions involving multiple non-telomeric loci across the CLL genome, including those with known copy number aberrations in CLL. Our data implicate diverse DNA repair mechanisms at play in CLL tumour initiation and progression, including C-NHEJ, A-NHEJ and SSA. These repair pathways provide potential therapeutic targets and combinations of therapeutic agents targeting these specific pathway components may effectively sensitise CLL B-cell clones with ongoing telomere dysfunction to improve patient outcomes.

## Supplementary information

Supplementary materials
