# Supplementary material for: Telomere fusions associate with coding sequence and copy number alterations in CLL
Source: Leukemia. 2019 Feb 22;33(8):2093–7. doi: 10.1038/s41375-019-0423-y (PMC6690834; doi:10.1038/s41375-019-0423-y)
Supplement: Supplementary file 1 — Supplementary materials [file 41375_2019_423_MOESM1_ESM.pdf]

Telomere fusions associate with coding sequence and copy number alterations in CLL

Laura Escudero[1], Kez Cleal[1], Kevin Ashelford[1], Chris Fegan[1], Chris Pepper[2], Kate Liddiard[1¶] and Duncan M. Baird[1¶]

[1] Division of Cancer and Genetics, School of Medicine, Cardiff University, Cardiff, United Kingdom;

[2] Brighton and Sussex Medical School, Sussex University, Brighton, United Kingdom.

[¶]Joint senior authors

Correspondence: Professor Duncan M. Baird, Division of Cancer Genetics, School of Medicine, Cardiff University, Heath Park, Cardiff CF14 4XN, United Kingdom; E-mail: bairddm@cf.ac.uk.

Supplementary Material:

Supplemental Subjects and Methods

Patient samples

Samples were obtained from CLL patients with informed consent following recruitment into the UK CLL4 (n=209), ARCTIC and ADMIRE (n=276) clinical trials, or consultation at the University Hospital of Wales (UHW; LREC: 13/WA/0346) (n=33) (1-3). Telomere Length at XpYp was analysed using Single Telomere Length Analysis (STELA) for all patient samples (4). Of the UHW samples, 26 patient samples had a Telomere Length (TL) <3.81Kb and 7 had a TL between 3.81-5.59Kb. TL measurements of the CLL4 and ARCTIC and ADMIRE samples were performed by Norris et al (manuscript in preparation) and only patients with TL<3.81Kb were used in subsequent analyses.

Isolation of peripheral blood mononuclear cells (PBMCs)

CLL blood samples from UHW were collected into 7.2mg K2-ethylenediaminetetraacetic acid. Lymphocytes were isolated from whole blood by density gradient centrifugation using Ficoll-Histopaque-1077 (Sigma-Aldrich). Patient PBMCs from the LRF CLL4, ARCTIC and ADMIRE clinical trials were obtained from the UK CLL Trials Biobank, University of Liverpool.

DNA extraction, telomere length analysis and fusion assay

Genomic DNA was extracted from UHW and LRF CLL4 trial CLL patient PBMCs containing >80% $CD19^+$ B cells or from ARCTIC and ADMIRE trials CLL patient PBMCs by standard RNase A, Proteinase K, phenol/chloroform extraction. TL analysis was performed using STELA for the 17p and XpYp chromosome ends as described previously (5). To measure 5p TL, a 5p-specific primer was designed (5p5: 5′-GGAGCAGCATTCTCTTCACCACAG-3′), the cycling conditions were adapted (T_m: 59°C) and 1.25ng/µL gDNA used. Telomere fusions were detected using an adaption of the fusion PCR assay that included the 17p; XpYp; 1p, 9p, 12p, 15q, XqYq and 2q13 interstitial locus for the 16p family of telomeres; 1q, 2q, 5q, 6q, 6p, 8p, 10q, 13q, 17q, 19p, 19q, 22q, the 2q13 interstitial locus and 21q for the 21q family of telomeres (4, 6) as illustrated in Supplementary

Figure 1. A 5p-specific primer (5p8: 5′-CCTCTACTAACCTTTAAGGCTGTG-3′) was designed in the 5p sub-telomeric sequence to target this chromosome end that is distal to *TERT*. For Southern blotting with a 5p-specific radiolabelled probe, the gel-purified product of the fusion primer 5p8 with 5p6: 5′-CGTAGAGGAGGGTGGAACCTC-3′ was used. For each CLL patient sample 100ng of gDNA was used per reaction and 10 replica telomere fusion PCR reactions were performed. From the total cohort of 276 CLL patient samples, the first 33 samples (15 from the UHW and 18 from the LRF CLL4 clinical trial) were screened with the radiolabelled probes combined in the following sequence: 5p+17p, XpYp+16p and 21q. The remaining 243 patient samples were initially screened using the 5p probe on its own to facilitate identification of 5p telomere fusions. Fusion frequency was calculated by dividing the total number of fusion events detected for each sample by the total number of diploid genomes used in the original PCR reaction.

Sequencing telomere fusion amplicons

For 9 CLL patient samples, 200-300 telomere-fusion PCR reactions were performed, generating 600-900 fusion amplicons per sample. Pooled fusion amplicons for each sample were purified using Agencourt AMPure XP beads. Verification of purification was assessed by Southern blotting. Fusion amplicons were subjected to paired-end Illumina HiSeq4000 PE100 sequencing at the Oxford Genomic Centre (CLL1 sample) and Beijing Genomics Institute, Hong Kong (samples CLL2-9).

Characterisation of intra- and inter-chromosomal telomere fusions

The mapping approaches taken to characterise telomere fusions and rare genomic recombination events were based on a novel pipeline previously developed (7), adapted to include the 5p sub-telomere, and delineated in Supplementary Figure 2. The human reference sequence employed was hg19, GRCh37. Identified telomere fusion sequences were visualised using the Broad Institute Integrative Genome Viewer (IGV)(8) and underwent a second round of alignment validation using Basic Local Alignment Search Tool (BLAST) to assess mapping

accuracy. Only unambiguously-aligned events were included in subsequent analyses and 68% of these included the actual fusion junction within a single read.

Intra-chromosomal telomere fusions were defined as paired-end sequence reads mapping to the same sub-telomeric sequence in the same orientation 5′-3′ towards the telomere repeat array. Inter-chromosomal telomere fusions were defined as paired-end sequence reads where at least one of the read pair was mapped to a defined sub-telomeric sequence and the other mapped to a locus on a different chromosome. Validated telomere fusion junctions were individually investigated to determine measures of microhomology (sequence identity) and deletion at contributing chromatids. Analyses of gene sets enriched within telomere fusions with the genome were performed using Gene Set Enrichment Analysis (GSEA, v5.2) and Molecular Signatures Database (MSigDB) (9).

Code availability

Relevant scripts can be downloaded via

https://github.com/nestornotabilis/GenomeResearch_2016_scripts.

Whole Genome Sequencing (WGS)

WGS was undertaken at BGI Technologies with Illumina HiSeq2000, using 30µg of $CD19^+$ CLL B-cells gDNA for 60x coverage of the tumour genome and 2µg of $CD3^+$ CLL T-cells gDNA for 30x coverage of the control.

Somatic SNVs were called using Mutect (10) and Somatic-Sniper (11) with default settings, and the intersection of these sets was taken to reduce the false positive rate for low frequency alleles. The variant allele frequency distribution was analysed at diploid regions of the genome identified from running cn.mops (12).

Statistics

All statistical analyses, including one- and two-tailed *t*-tests, ANOVA tests and Chi-square analyses were performed using GraphPad software, including Prism 6.

Availability of data and material

The sequencing data is deposited in NCBI under a BioProject ID: PRJNA459488 and available at http://www.ncbi.nlm.nih.gov/bioproject/459488

Supplementary figures

A

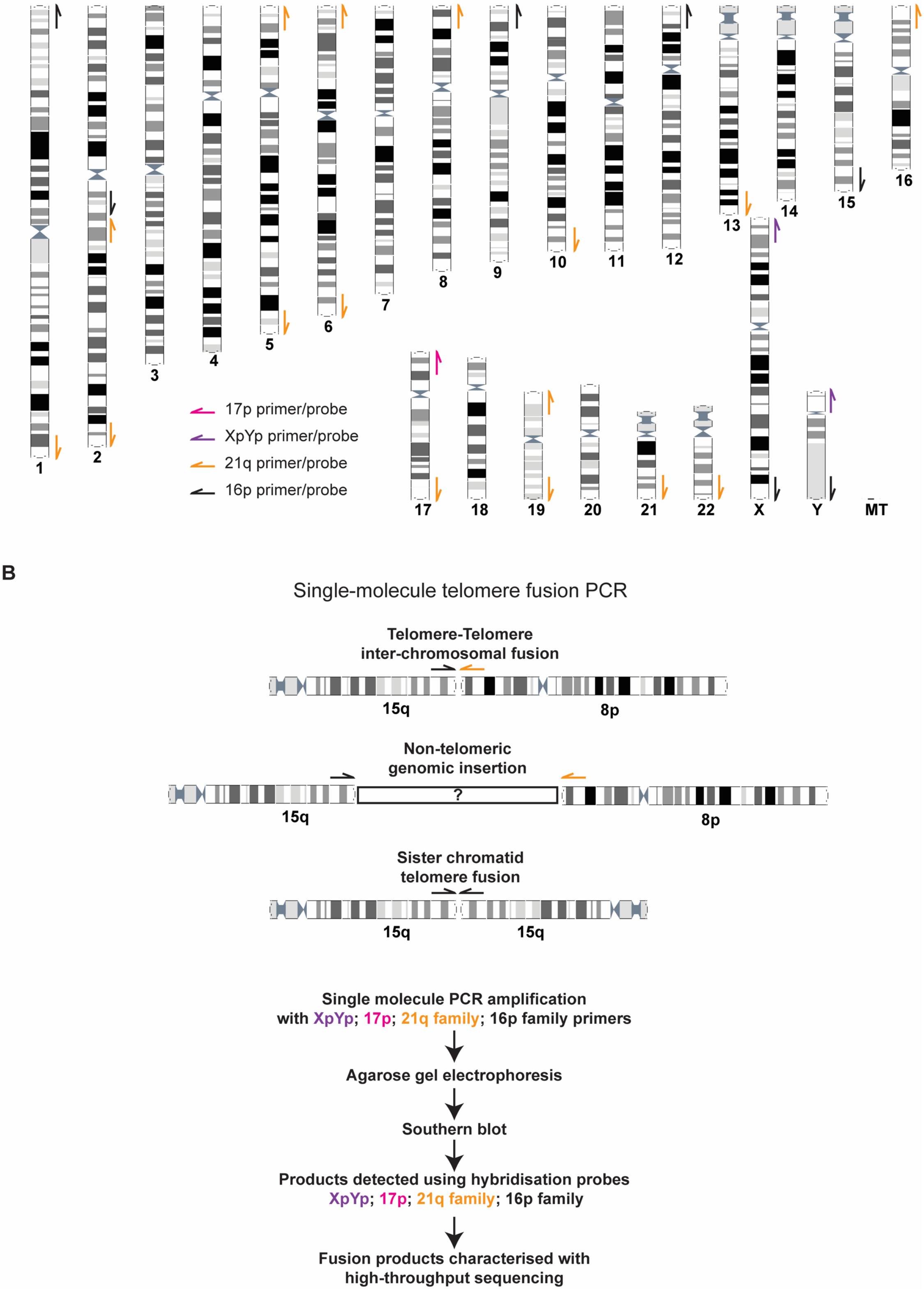

Supplementary Figure 1. Illustrating the single-molecule telomere fusion assay. (A) showing the distribution of telomere-adjacent PCR primers and accompanying probes used for single-molecule telomere fusion analysis. The unique telomeres of XpYp and 17p are shown in purple and red respectively, with the 16p and 21q families of telomers shown in black in orange. **(B)** detailing the work flow for telomere fusion analysis and characterisation with high-throughput sequencing.

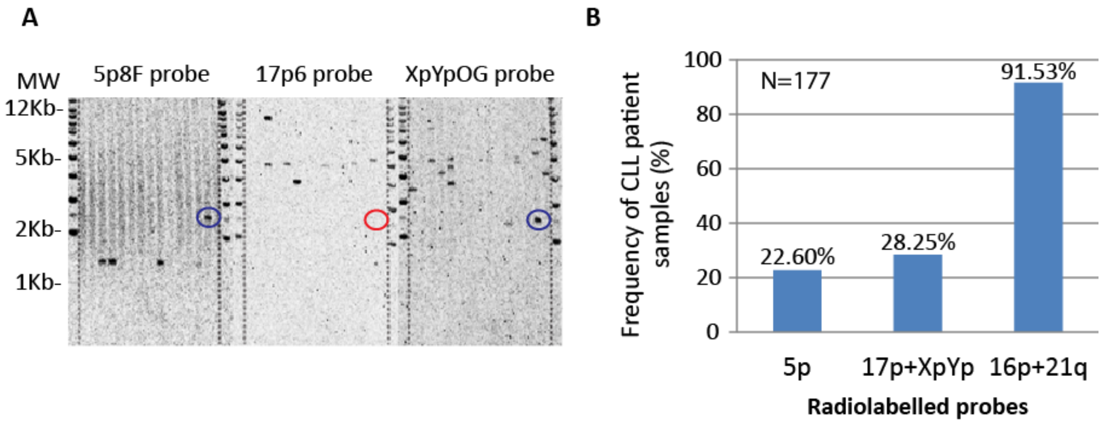

Supplementary Figure 2. Detection of 5p fusions in CLL patient samples.

(A) Example of a 5p-XpYp telomere fusion event in a CLL patient sample. Southern blots were hybridised with the 5p8F, 17p6 and XpYp, respectively. Blue circles highlight the fusion event and the red circle indicates the absence of the event. **(B)** Proportion of CLL patient samples for which 5p, 17p+XpYp and/or 16p+21q telomere fusions were detected.

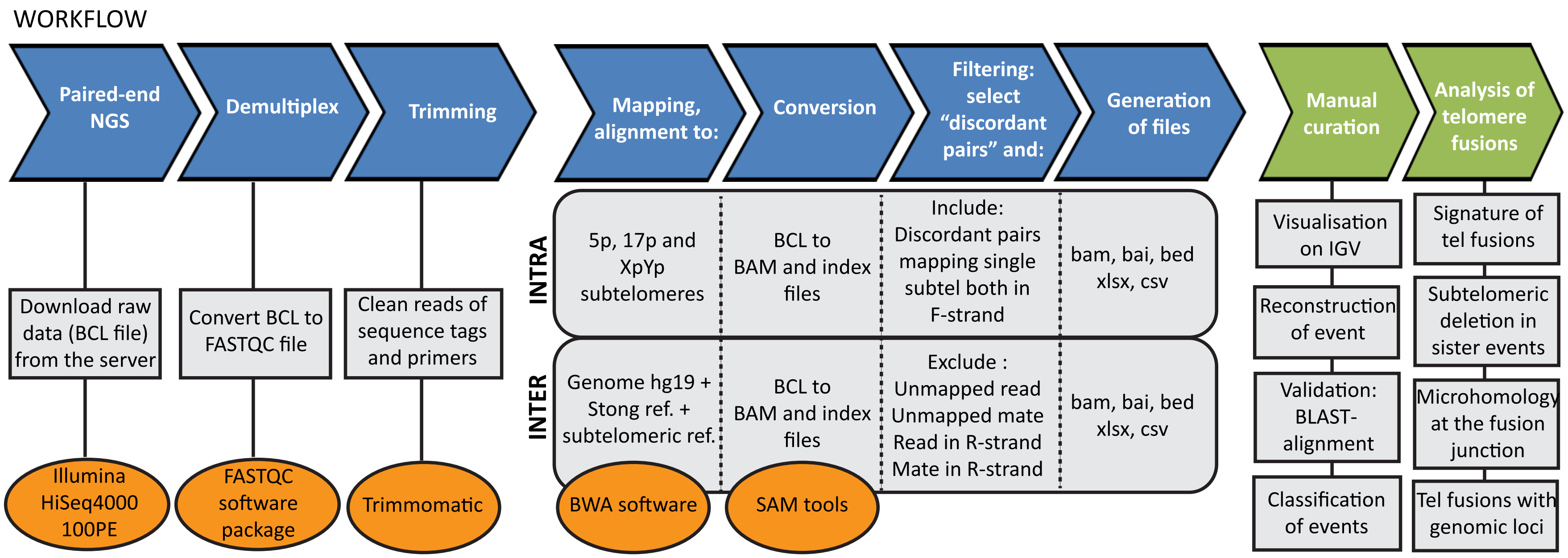

Supplementary Figure 3. Workflow for the NGS analysis of telomere fusion amplicons.

Pipeline for the detection of telomere fusion events using bioinformatics tools, followed by manual curation and downstream analysis for each CLL patient sample. Sequencing data of telomere fusion amplicons from 9 CLL patient samples were obtained from BGI Tech after they performed HiSeq4000 PE NGS. Data handling and QC were performed following intra- and inter-chromosomal mapping strategies. Finally, manual curation and downstream analysis were performed.

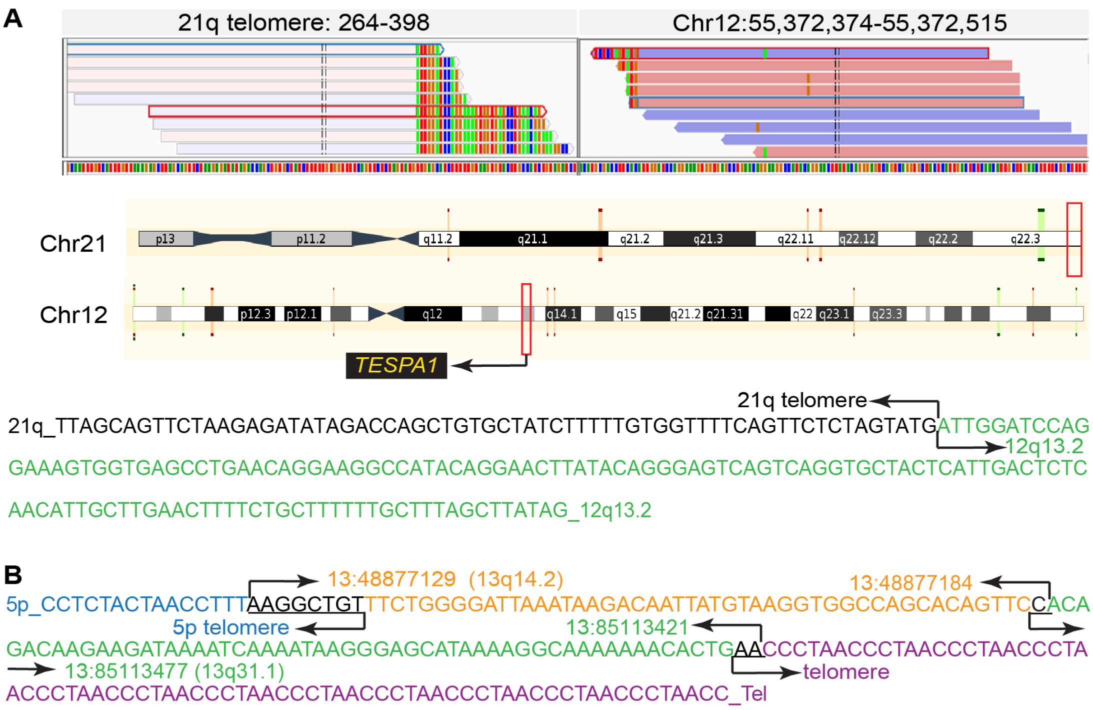

Supplementary Figure 4. Examples of telomere fusion events sequenced from CLL patients. (A) Illustration of a 21q telomere fusion with a non-telomeric genomic locus at Chr12q13.2 Paired-reads and fusion junction visualised using the tool Integrative Genome Viewer (IGV). The colours represent the soft-clipping that may identify a sequence aligning elsewhere in the genome. Location of the reads shown in Ensembl (Gene identified *TESPA1*: Thymocyte expressed, positive selection associated 1; ENSG00000135426) and sequence of the fusion event. All events were sequence-verified and BLAST-authenticated. (B) Example of a complex fusion event with 4 distinct loci (represented with different colours) including Chr13q14.2 that is frequently deleted in CLL. Microhomology in black and underlined to indicate short sequences of DNA shared at the fusion junction.

5:14788 ←

A 5p_GGGCTTGTCTAGTTCTGCCCAGGCATGCTGGAGCAAGCTAGGATAACTGATTTGGAATACACACACACAC

ACTCtaaattccaacaagtctacagactttctagtgtgatcccacaagccccaccgggaggcatggcccagcacttgcaaatgaatctccacga

→ 6:17847350 (*KIF13A*)

ggcgtggcaatcttttctttgaaacctgct_Chr6p22.3

5:14786 ←

B 5p_TCTGCCCAGGCATGCTGGAGCAAGCTAGGATAACTGATTTGGAATACACACACACACTCACctaccatcctt

→ 5:27233931

atatttacttacaatttacatctttaccaagaaaattttctttaataattaatccatctttaattccaaatatgtgtgtatgcaaatgatatactagcc

atattatcatgccta_Chr5p14.1

5:15226 ←

C 5p_CCTCTACTAACCTTTAAGGCTGTGAACCCTGTAATCTAGGTATCAGGCTGGCTTTTCTCAtccctgcagttgaact

→ 2:118579266 (*DDX18*)

cattgttaagttaaggttcatgcccaggaatggtaagcgttcatctgcttgtttctgccattatttcactagaggtttattgtagctgttacggactca

gtggtgacagccac_Chr2q14.1

D 5p_ATGGCATGCACTATGGGCTGGGGCTTGTCTAGTTCTGCCCAGGCATGCTGGAGCAAGCTAGGATAACTGA

5:14794 ←

TTTGGAATACACACACACccacaaattagtaccaggacattgtgtcctgttaaacttactacaaccatgcaaagatcgatattattaaccc

→ 3:118534700

taccttagaattaagggaactgatcatcagaatggtt_Chr3q13.32

5:15208 ←

E 5p_CCTCTACTAACCTTTAAGGCTGTGAACCCTGTAATCTAGGTATCAGGCTGGGTTTTCTCAGAGTGCTGTTGG

GAATGAgagagaggttggcagaaggagaagttagattcgagaggtaagagagactcaattagtcgcgggagggagcaccacgggaagcat

→ 1:219990571 (*SLC30A10*)

gaggaggaatggagcagcctc_Chr1q41

5:13905 ←

F 5p_AACTGACAAGGGTATTCGATTTTTTCTGTGGCCGCCCAAAGTGCTGGGACTACAGGCGCCTGCCACCACAG

CCGGCTgtacagcatgttactctactgagtagagtaggcaactgtatttgtacatctaaacgtagaaaaaatacagtaaaaatatggtattata

→ 22:42986484 (*POLDIP3*)

atgttatg_Chr22q13.2

5:13898 ←

G 5p_TATTCGATTTTTTCTGTGGCCGCCCAAAGTGCTGGGACTACAGGCGCCTGCCACCACAGCCGGCTAATTTTT

gtatttttagtagagatgacgttttgccatgttggtcaggctggtcttgaactcccaacctcaggtgatccacctgccttggtctcccaaagtgttgg

→ 19:14303227 (*LPHN1*)

gattacaggcgt_Chr19p13.12

Supplementary Figure 5. Long tracts of microhomology at inter-chromosomal fusion junctions with genomic loci. Examples of 5p inter-chromosomal fusions with genomic loci with different usage of microhomology (MH). (A) 5p-chr6p22.3, MH=18bp (CLL3), (B) 5p-chr5p14.1 MH=18 (CLL3), (C) 5p-chr2q14.1 MH=13bp (CLL4), (D) 5p-chr3q13.32 MH=12 (CLL9), (E) 5p-chr1q41 MH=9bp (CLL8), (F) 5p-chr22q13.2 MH=6bp (CLL2) and (G) 5p-chr19p13.12 MH=6bp (CLL8). 5p subtelomeric sequence in capitals, genomic loci in green and microhomology at the fusion junction underlined.

A Tel_TTAGGGTTAGGGTTAGGGTTAGGGTTAGGGTTAGGGTTAGGGTTAGGGTTAGGGTTAGGGTTAGGGTT

Telomere ←

AGGGTTAGGGTTAGGGT***a***agtgggaagaagaaagagaggaagtaaagtttaattatgcctttttgggttgaggtgatgatggaggtg

→ MT:14120 (***MT-ND5***)

gagatttggtgctgtgaaattgtttt_ChrMT

MT-ND5: Mitochondrially encoded NADH dehydrogenase 5, ENSG00000198786, exon 1/1, pc

B 16p_GTTTAAACTACATGCAGGAACAGCAAAGGAAATCCGGCAAATTTGCGCAGTCATTCTCAACACCGGC

16:61426 ←

CATGCAGCAAAATCATtactattctgcctagcaaactcaaactacgaacgcactcacagtcgcatcataatcctctctcaaggacttc

→ MT:11729 (***MT-ND4***)

aaactctgctcccactaatagctttttgatgactt_ChrMT

MT-ND4: Mitochondrially encoded NADH dehydrogenase 4, ENSG00000198886, exon 1/1, pc

C 16p_GCCCTGGGCACTATGGTTTTGAGCTTGTACCCAGCGCTGCTTTTGCCTTGCTCTGTGACCCCAGGCAA

16pfam; 16:64957-65056 ←

GCTGCCTCACCTCTCTGGGCCAGTTTCCCCAT(...)(...)atactggcattttgtagatgtggtttgtctatttctgtatgtctccat

→ MT:9926-10025 (***MT-COA***, ***MT-TG***)

ctattgatgagggtcttactcttttcgtataaatagtaccgttaacttccaa_ChrMT

MT-CO3: Mitochondrially encoded cytochrome c oxidase III, ENSG00000198938, exon 1/1, pc
MT-TG: Mitochondrially encoded tRNA glycine, ENSG00000210164, exon 1/1, known Mt tRNA

D 5p_CACACACACACTCACGTATATATATAAATCGTGGATAGCAATCTTATAGTAAGAAATTGTGACTTTTTA

5:14701-14800 ←

TAATCTTTGAAGAACTTTCCCATTTCTCACA(...)(...)taaagctaaaactcacctgagttgtaaaaaactccagttgacacaa

→ MT:968-1067 (***MT-RNR1***)

aatagacaacgaaagtggctttaacatatctgaacacacaatagctaagaccca_ChrMT

MT-RNR1: Mitochondrially encoded 12S RNA, ENSG00000211459, exon 1/1, known Mt rRNA

Supplementary Figure 6. Telomeric fusion with mitochondrial DNA. Four examples of telomeric fusions with mtDNA. Fusion junction mapped for events **A** (CLL4) and **B** (CLL9), unmapped for **C** (CLL8) and **D** (CLL9). Mitochondrial DNA was represented in lowercase and pink, telomeric sequence was in capitals. Microhomology at the fusion junction was underlined and the insertion in highlighted in bold. Coordinate of the FJ and disrupted genes are indicated (pc: protein coding). Telomere fusions with metabolic genes encoded in the mitochondrial genome include 12S RNA (*MT-RNR1*), cytochrome c oxidase III (*MT-CO3*), tRNA glycine (*MT-TG*), NADH dehydrogenase 4 (*MT-ND4*) and NADH dehydrogenase 5 (*MT-ND5*).

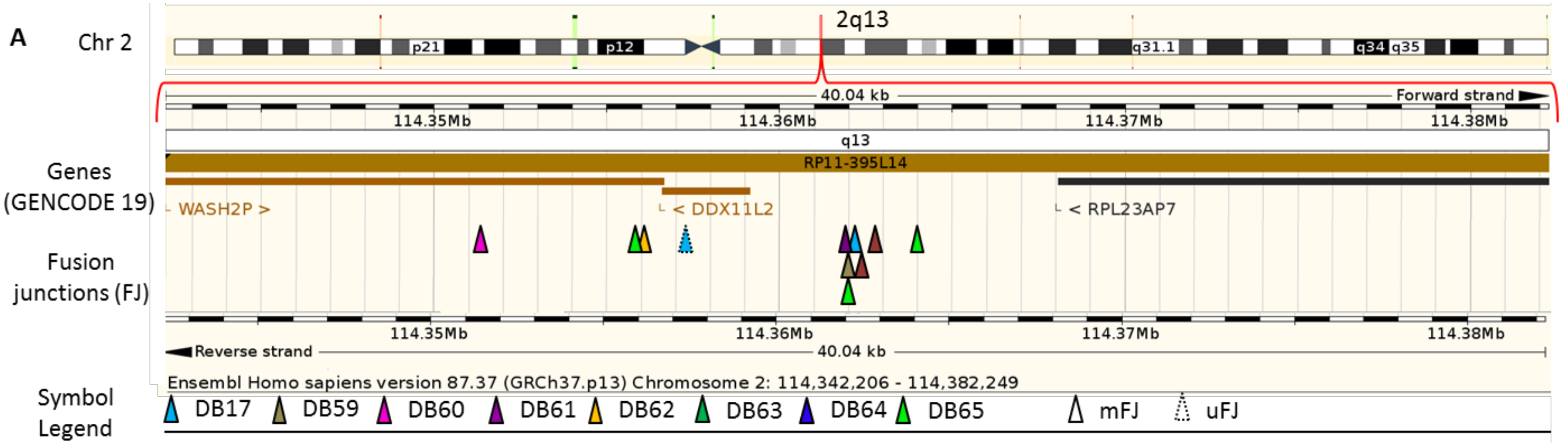

B

Telomere

Tel_GTTAGGGTTAGGGTTAGGGTTAGGGTTAGGGTTAGGGTTAGGGTTAGGGTT<u>AGGG</u>cgggcagcggccctgcctcctacccttgcgcctcatgaccagctt_2q13

2:114356070

C

9:12122

16p_GGCCAGAAGATGAACAATGGCCCATCCCACTCTAGGCATGGCTCCTCTCCACAGGAAAACTCCACTCCAGTGCTCAGCTTGCACCCTGGCACAGGCCAGCAGTTG CTGGAAGTCAG<u>ACACC</u>cgcctgctggcagctgggcacactgccgggccctcttgctcgcattgtcgtggctgcacgccacatgcaggcacatggggactacgcagggccctcttgctcccggtgtgacggctggcgt cccatattggccacct_2q13

2:114361894

D

16:61214 2:114363893

16p_TCTGATGGTAATCAGTTAACAAATTAAACATATTTATTTTATACTTTTAAggcttaagaattattccaagctaattattttgaaaaaaaatgctaacaaaggaagttgtgaaaatagagaagtt acttgtgtaaggaaaatttacatctataaaggaaatcaccatttaaaagctacctctctcgacaccaag_2q13

Supplementary Figure 7. Telomeric fusions with the ancestral telomere at 2q13.

(A) Location of fusion junctions on 2q13 visualised in Ensembl. Arrow heads indicate fusion junctions (continuous line for mapped FJ and dotted line for unmapped FJ for which the read location is represented) and each CLL patient sample is represented with a different colour. Gene legend: gold (protein coding, merged Ensembl/Havana) black (non-protein coding, pseudogene). **(B-D)** Three examples of telomeric (capital letters)-2q13 (green) fusions represented (from CLL5, CLL2 and CLL8, respectively). Microhomology at the fusion junction was underlined.

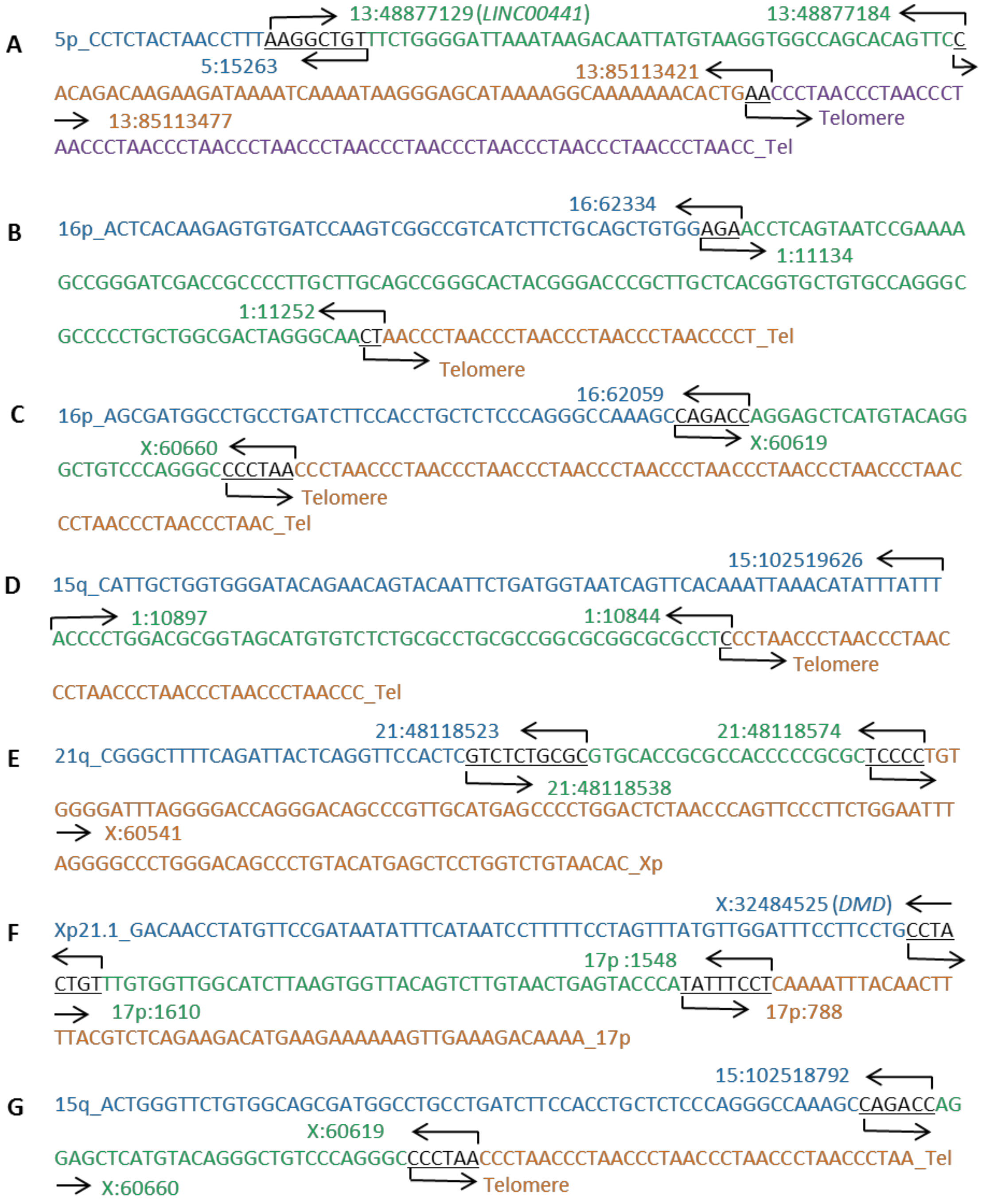

Supplementary Figure 8. Complex telomere fusion events are detected in patient CLL-B cells. Complex telomere fusions obtained from the inter-chromosomal analysis of 9 CLL patient samples (n=7). (A) 5p-Chr13q14.2(LINC00441)-chr13q31.1-Tel, (B) 16p-1p-ccctaa, (C) 16p-Xp-Tel, (D) 15q(16p)-16p-Tel, (E) 21q+21q+Xp, (F) Xp21.1(DMD)-17p-17p and (G) 15q(16p)-Xp-cccta. Microhomology at the fusion junctions was highlighted in black and underlined, coordinates noted. Different colours were used to distinguish the distinct locations involved in the fusion event (loc1=blue, loc2=green, loc3=brown, loc4=purple).

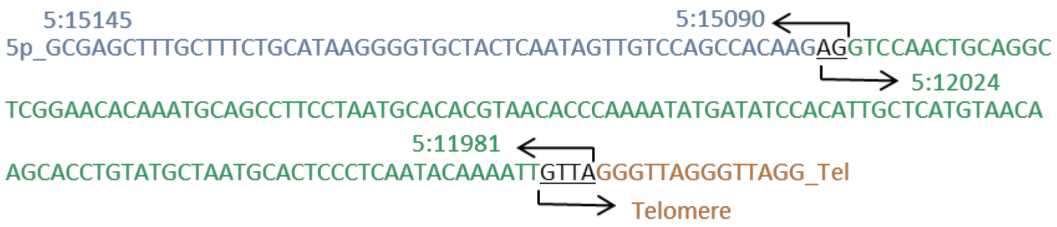

Supplementary Figure 9. Sister-chromatid fusion event detected from the inter-chromosomal analysis. 5p-5p intra-chromosomal fusion event potentially originated after a 5p-telomere fusion event (CLL9). Microhomology at the fusion junction is highlighted in black and underlined. Different colours used to distinguish the distinct sections of the fusion event.

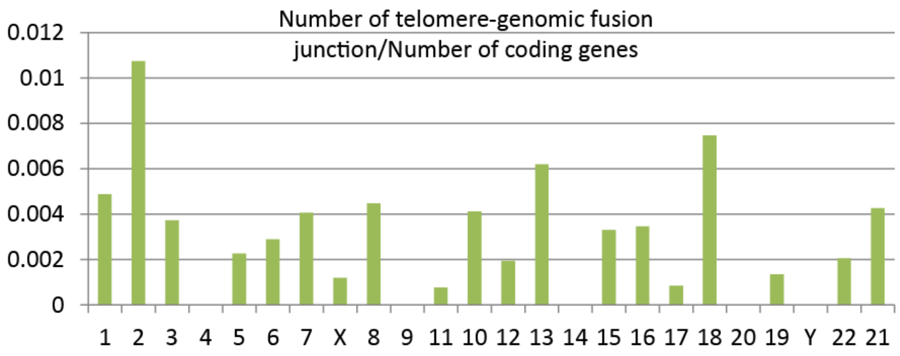

Supplementary Figure 10. Characterisation of fusion junctions.

Number of inter-chromosomal telomere-genomic fusion junctions per coding gene content for each chromosome ordered by length (gene content and size obtained from Ensembl).

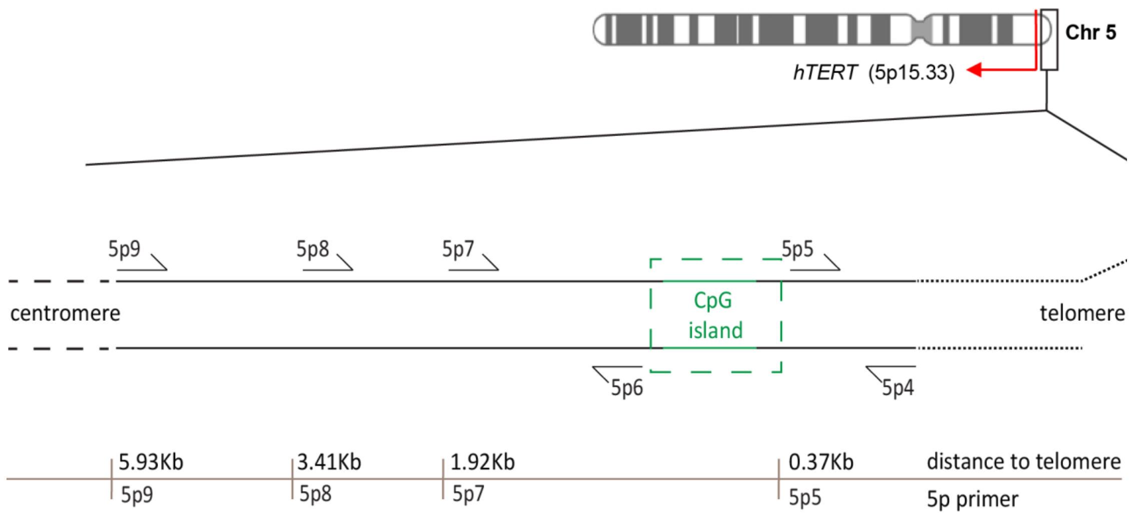

Supplementary Figure 11. CpG island at the 5p chromosome end. Chromosome 5. The 5p telomere is highlighted by a black box and the location of the *hTERT* gene locus is indicated by a red arrow. A representation of the 5p subtelomeric region with the location of 5p primers and distance to the start of the telomere. GC-rich DNA sequence is highlighted by a green box and highlights primer combinations at each side.

Supplementary tables

Supplementary Table 1. Number and proportion of CLL patients for each category of fusions.

			ALL				HOSPITAL				LRF CLL4				ARCTIC and ADMIRE			
**CATEGORY**	**#FUSIONS**	**FREQUENCY**	#	%	G#	G%	#	%	G#	G%	#	%	G#	G%	#	%	G#	G%
**NONE**	0	$0.00E^{+00}$	**78**	28.26	78	28.26	**11**	33.33	11	33.33	**14**	35.90	14	35.90	**53**	25.98	53	25.98
	1	$6.00E^{-06}$	**72**	26.09			**4**	12.12			**13**	33.33			**55**	26.96		
**LOW**	2	$1.20E^{-05}$	**41**	14.86	143	51.81	**3**	9.09	11	33.33	**5**	12.82	20	51.28	**33**	16.18	112	54.90
	3	$1.80E^{-05}$	**30**	10.87			**4**	12.12			**2**	5.13			**24**	11.76		
	4	$2.40E^{-05}$	**22**	7.97			**5**	15.15			**2**	5.13			**15**	7.35		
**MEDIUM**	5	$3.00E^{-05}$	**13**	4.71	40	14.49	**1**	3.03	8	24.24	**2**	5.13	4	10.26	**10**	4.90	28	13.73
	6	$3.60E^{-05}$	**5**	1.81			**2**	6.06			**0**	0.00			**3**	1.47		
**HIGH**	>7	$4.20E^{-05}$	**15**	5.43	15	5.43	**3**	9.09	3	9.09	**1**	2.56	1	2.56	**11**	5.39	11	5.39
		**TOTAL**	**276**	100	276	100	**33**	100	33	100	**39**	100	39	100	**204**	100	204	100

Number of telomere fusion events and frequency of fusions detected from 10 fusion PCR reactions, 100ng gDNA/reaction.

All CLL patient samples presented TL≤3.81Kb except for 7 patient from the UHW with TL:3.81-5.59Kb.

column indicates the total number of events

% indicates the % of the total

G# indicates the subgroup total number of events

G% indicates the subgroup % of the total

Supplementary Table 2. Description of potential 5p fusions detected in CLL patient samples.

CATEGORY	#FUSIONS	FREQUENCY	5p FUSIONS: TYPE & SIZE (Kb)	ID-CLL
**LOW**	1	6.00E-06	5p-21q (~2.75)	68
**LOW**	1	6.00E-06	5p-5p (~0.8)	29
**LOW**	1	6.00E-06	5p-16p/21q (~0.5)	144
**LOW**	1	6.00E-06	5p-16p/21q (~2.6)	182
**LOW**	1	6.00E-06	5p-5p (~1.6)	198
**LOW**	2	1.20E-05	5p-16p/21q (~2.75)	107
**LOW**	2	1.20E-05	5p-5p (~1.75)	86
**LOW**	2	1.20E-05	5p-5p (2kb)	142
**LOW**	2	1.20E-05	5p-5p (~1.75)	153
**LOW**	2	1.20E-05	5p-5p (1.75)	160
**LOW**	2	1.20E-05	5p-5p (~1.75); 5p-5p (~4.1)	269
**LOW**	2	1.20E-05	5p-5p (~1.75); 5p-16p/21q (~2.75)	274
**LOW**	3	1.80E-05	5p-16p/21q (~2.75)	236
**LOW**	3	1.80E-05	5p-5p (~1.75)	76
**LOW**	3	1.80E-05	5p-5p (~2.75); 5p-5p (~2.9)	117
**MEDIUM**	4	2.40E-05	5p-16p/21q (~2.75) x2	60
**MEDIUM**	4	2.40E-05	5p-16p/21q (~2.75)	217
**MEDIUM**	4	2.40E-05	5p-5p (~1.6)	232
**MEDIUM**	4	2.40E-05	5p-5p (~1.75)	233
**MEDIUM**	4	2.40E-05	5p-5p (~0.25); 5p-5p (~1.2)	243
**MEDIUM**	4	2.40E-05	5p-5p (~1.75)	255
**MEDIUM**	4	2.40E-05	5p-5p (~0.75)	271
**MEDIUM**	5	3.00E-05	5p (~2.75)	113
**MEDIUM**	5	3.00E-05	5p-16p/21q (~2.75)	120
**MEDIUM**	5	3.00E-05	5p-5p (~3.7); 5p-5p (~1.2)	235
**MEDIUM**	5	3.00E-05	5p-5p (~0.75)	246

**MEDIUM**	5	$3.00E^{-05}$	5p-17/XpYp (~2.0)	272
**MEDIUM**	6	$3.60E^{-05}$	5p-16p (~0.4)	59
**MEDIUM**	6	$3.60E^{-05}$	5p-17/XpYp (~3.5)	112
**HIGH**	7	$4.20E^{-05}$	5p-16p/21q (~2.75)	219
**HIGH**	7	$4.20E^{-05}$	5p-17/XpYp (~6.5)	253
**HIGH**	8	$4.80E^{-05}$	5p-5p (~0.5)	108
**HIGH**	8	$4.80E^{-05}$	5p-5p (~0.25); 5p-16p/21q (~2.75)	121
**HIGH**	8	$4.80E^{-05}$	5p-5p (~1.75); 5p-16p/21q (~1.0)	215
**HIGH**	8	$4.80E^{-05}$	5p-5p (~0.75)	251
**HIGH**	10	$6.00E^{-05}$	5p-21q (~2.75); 5p-5p (~4kb)	65
**HIGH**	12	$7.200E^{-05}$	5p-5p (~1.75)	226
**HIGH**	~20	$\sim 1.20E^{-04}$	5p-YpYp (~2.5)	41
**HIGH**	~20	$\sim 1.20E^{-04}$	5p-21q (~5.5); 5p-21q (~7.5)	70
**HIGH**	~20	$\sim 1.20E^{-04}$	5p-5p (~1.6); 5p-5p (~2.3); 5p-16p/21q (~2.75)	118

Supplementary Table 3. Validated telomere fusion events identified from a panel of 9 CLL patient samples.

Type	C*	Type of telomere fusion event	Example	Total (N)	Total (%)
**00**	-	Telomere-Telomere (head-to-head fusion without sub-telomeric sequence identified)	TTAGGG-CCCTAA	16	**1.75**
**0**	-	Sub-telomere-Telomere	5p-CCCTAA<br>5p-TTAGGG	360	**39.39**
**1**		Intra-chromosomal fusion event (sister-chromatid)	5p-5p	49	**5.36**
**2T**		Inter-chromosomal: two distinct **T**elomeres	5p-Xp;	343	**37.53**
**2A**		Telomere fusion with **A**ncestral telomere 2q13	5p-Chr2q13	11	**1.20**
**2G**		Telomere fusion with **G**enomic loci	5p-Chr5p14.1	75	**8.21**
**2C**		**C**omplex telomere fusion event involving multiple loci	5p-Chr13q14.2-Chr13q31.1-Tel	7	**0.77**

**2M**		Telomere fusion with Mitochondrial DNA)	5p-ChrM	4	**0.44**
**1/2T**		Intra or Inter: fusion of the 16p or 21q family of telomeres	16p-16p;<br><br>21q-21q	49	**5.36**
			**TOTAL**	914	**100%**

*Colour representing each category on the Circos plots. Event types 00 and 0 not represented. Complex telomere fusion represented according to each locus involved.

Supplementary Table 4. Summary of validated events resulting from intra/inter-chromosomal fusion analysis.

CLL patient sample	Total events	Tel-Tel (#00)	Subtel-Tel (#0)	Intra (#1)	Intra/Inter (#1o2)	Inter (#2T)	2q13 (#2A)	Genomic (#2G)	Complex (#2C)	Mt DNA (#2M)
**CLL1**	160	0	88	15	21	29	2	5	0	0
**CLL2**	136	3	96	3	3	19	1	9	2	0
**CLL3**	142	0	51	5	9	64	1	8	4	0
**CLL4**	47	0	17	0	1	15	1	12	0	1
**CLL5**	166	4	36	5	4	111	1	4	1	0
**CLL6**	70	4	54	6	2	3	0	1	0	0
**CLL7**	33	1	6	7	4	6	0	9	0	0
**CLL8**	32	4	2	1	0	4	3	17	0	1
**CLL9**	128	0	10	7	5	92	2	10	0	2
**TOTAL**	**914**	**16**	**360**	**49**	**49**	**343**	**11**	**75**	**7**	**4**
**TOTAL (%)**	**100**	**1.75**	**39.39**	**5.36**	**5.36**	**37.53**	**1.20**	**8.21**	**0.77**	**0.44**
**Colour-code Circos plots**		-	-							

Supplementary Table 5. Description of genomic loci that fuse with dysfunctional telomeres.

FEATURE	Unmapped fusion junction	Mapped fusion junction (mFJ)	All	mFJ	hg19
**PC INTRON**	*EIF2B5, C8A, ORAI1, DCAF6, RDH8, SLC39A12, ZNF678, TMEM63C*	*POLDIP3, TESPA1, CD8A, KIF26B, RORA, EVI5, FGGY, ZNF254, LPHN1, FAM78B, CSMD1, KIF13A, PCNXL2, NTF3, BEND7, FTO, SLC30A10, C6orf123, VPS13D, ECE1, PTCD3, HTR7, DGKB, NOX5, HDDC2, SHQ1, DMD, TBC1.*	43.9%	51.85%	
**PC EXON**	*KIF13A, MIP, RALYL, MT-CO3, MT-RNR1, MT-TG*	*DDX18, MT-ND4, MT-ND5*	9.76%	5.56%	
**TOTAL PC**			53.66%	57.41%	41.8%
**INTERGENIC**	-	-	40.24%	37.04%	
**NPC**	-	*CTC-575N7.1*	1.22%	1.85%	
**LINC**	*LINC01090*	*LINC00441*	2.44%	1.85%	
**PSEUDOGENE**	*RP11-353N4.6*	*RP11-520H11.1*	2.44%	1.85%	

**CFS**	**FRA12E** (12q24.31), **FRA1F** (1q21.2), **FRAK1** (1q32.1), **FRA2H** (2q32.1), **FRA3C** (3q27.1), **FRA1B** x2 (1p32.2, 1p32.3),	**FRA10D** (10q22.1), **FRA15A** (15q22.2), **FRA18B** (18q21.32), **FRA1D** (1p22.1), **FRA1B** (1p32.1), **FRA1I** (1q44), **FRA3D** (3q25.2), **FRA5E** (5p14.1).	18.30%	14.8%	14.9%
**ALU**	**AluY** x3 (2q32.1, 17q24.2, 8p12), **AluSx3** (1q24.2), **AluJr** x2 (10p12.33, 19p13.2), **FLAM_A** (1p32.3)	**AluY** (15q22.2), **AluSx3** x2 (19p12, 19p13.12), **AluJr4** (1p22.1), **AluJb** (22q13.2).	14.63%	9.25%	11%

PC: protein coding; NPC: non-protein coding; LINC: long intergenic non-coding RNA; CFS: common fragile site; ALU: Alu element.

Supplementary Table 6. List of human genes with validated fusion junction submitted to DAVID and GSEA.

ENSEMBL	ENTREZ	Gene	Gene Name
**ENSG00000048707**	55187	*VPS13D*	vacuolar protein sorting 13 homolog D
**ENSG00000067208**	7813	*EVI5*	ecotropic viral integration site 5
**ENSG00000069667**	6095	*RORA*	RAR related orphan receptor A
**ENSG00000072071**	22859	*LPHN1 (ADGRL1)*	Latrophilin (adhesion G protein-coupled receptor L1)
**ENSG00000088205**	8886	*DDX18*	DEAD-box helicase 18
**ENSG00000100227**	84271	*POLDIP3*	DNA polymerase delta interacting protein 3
**ENSG00000111906**	51020	*HDDC2*	HD domain containing 2
**ENSG00000117298**	1889	*ECE1*	endothelin converting enzyme 1
**ENSG00000121749**	64786	*TBC1D15*	TBC1 domain family member 15
**ENSG00000132300**	55037	*PTCD3*	pentatricopeptide repeat domain 3
**ENSG00000135426**	9840	*TESPA1*	thymocyte expressed, positive selection associated 1
**ENSG00000135749**	80003	*PCNX2*	pecanex homolog 2 (Drosophila)
**ENSG00000136267**	1607	*DGKB*	diacylglycerol kinase beta
**ENSG00000137177**	63971	*KIF13A*	kinesin family member 13A
**ENSG00000140718**	79068	*FTO*	FTO, alpha-ketoglutarate dependent dioxygenase
**ENSG00000144736**	55164	*SHQ1*	SHQ1, H/ACA ribonucleoprotein assembly factor
**ENSG00000146521**	26238	*C6orf123 (LINC01558)*	chromosome 6 open reading frame 123/ long intergenic non-protein coding RNA 1558
**ENSG00000148680**	3363	*HTR7*	5-hydroxytryptamine receptor 7
**ENSG00000153563**	925	*CD8A*	CD8a molecule
**ENSG00000162849**	55083	*KIF26B*	kinesin family member 26B
**ENSG00000165626**	222389	*BEND7*	BEN domain containing 7
**ENSG00000172456**	55277	*FGGY*	FGGY carbohydrate kinase domain containing
**ENSG00000183117**	64478	*CSMD1*	CUB and Sushi multiple domains 1
**ENSG00000185652**	4908	*NTF3*	neurotrophin 3
**ENSG00000188859**	149297	*FAM78B*	family with sequence similarity 78 member B
**ENSG00000196660**	55532	*SLC30A10*	solute carrier family 30 member 10
**ENSG00000198786**	4540	*MT-ND5*	NADH dehydrogenase, subunit 5 (complex I)
**ENSG00000198886**	4538	*MT-ND4*	NADH dehydrogenase, subunit 4 (complex I)

**ENSG00000198947**	1756	*DMD*	dystrophin
**ENSG00000213096**	9534	*ZNF254*	zinc finger protein 254
**ENSG00000255346**	79400	*NOX5*	NADPH oxidase 5
**ENSG00000231473**		LINC00441	long intergenic non-protein coding RNA 441
**ENSG00000251680**		CTC-575N7.1	novel antisense; non protein coding
**ENSG00000262987**		RP11-520H11.1	unprocessed pseudogene

Supplementary Table 7. List of genes with validated fusion junction associated with CLL and B cell or other oncogenes.

Gene	Gene name	Evidence in B lymphocytes or CLL-B cells
HTR7	Serotonin receptor	Genes found overexpressed in CD38[+] CLL cells (13). High levels of CD38 are associated with shorter overall survival (13, 14).
KIF26B	Kinesin family member 26B	
LPHN1	Latrophilin 1	
CD8A	CD8a molecule	Expressed in cytotoxic T lymphocytes but aberrant expression has been reported in low frequencies in patient CLL-B cells and carries an adverse prognostic impact in the disease (15).
RORA	RAR Related Orphan Receptor A	Involved in lymphocyte development and inflammatory responses and has been found over expressed in CLL among other cancers (16-19). *RORA* is also a very large CFS gene (within FRA15A 15q22.2) susceptible to genomic instability and inactivated in many tumours (20).
TESPA1	Thymocyte Expressed, Positive Selection Associated 1	Expressed in T and B lymphocytes and regulates the inositol 1,4,5-trisphosphate (IP_3R) calcium-dependent activation of signalling pathways playing an important role modulating immune function (21).
DMD	Dystrophin	Expressed at low but stable level in B cells and upregulated in unmutated CLL cases which associated with shorter survival (22, 23).
NOX5	NADPH Oxidase 5	Expressed in B cells in Hairy Cell Leukaemia (HCL)(24).<br>NOX-related damage, like reactive oxigen species (ROS), has been associated to the initiation and progression of haematopoietic malignancies (25).
FTO	Fat mass and obesity associated gene	The lipid metabolism gene *FTO* has been implicated in cancer cell metabolism (26). Unlike normal B cells, CLL cells present an altered lipid metabolism. Similarly to adipocytes and myocytes, they store lipids in vacuoles, produce energy from free fatty acids (FFA) and express genes related to the lipid metabolism (22, 27, 28).
NTF3	Neurotrophin 3	B cells are a source of neurotrophins that provide protective autoimmunity in the damaged nervous system; however, they

		express the neurotrophins NGF and BDNF but do not seem to express NTF3 and trkB (29).
EVI5	Ecotropic viral integration site 5	Regulator of cell cycle progression and cytokinesis. It has been suggested to prevent exhaustion in pre-leukemic stem cells in Runx1-deficient mouse and to cooperate with BCL6 (B cell lymphoma 6) transcription factor in B and T-cell lymphomas (30, 31). In addition, deletion of 1p22 comprising *EVI5* has been identified in over 20% of patients with multiple myeloma (MM) and low expression of this gene associates with worse prognosis in early stage patients (32).

Supplementary Table 8. Gene set overlaps found between a list of genes with validated fusion junction disrupted by inter-chromosomal telomere fusions and the Molecular Signatures Database (MSigDB v. 5.2) using Gene Set Enrichment Analysis (GSEA v.5.0; Broad Institute).

MSigDB collection	MSigDB gene set name	Number of genes in gene set (K)	Description of gene set	Number of gene list genes in overlap (k)	k/K	*p*-value	False Discovery Rate (FDR) FDR *q*-value
**C2**	PEPPER_CHRONIC_LYMPHOCYTIC_LEUKAEMIA_UP	33	Genes up-regulated in CD38+ [GeneID=952] CLL (chronic lymphocytic leukaemia) cells.	3 (HTR7, LPHN1, KIF26B)	0.091	$1.5\ e^{-6}$	$7.07\ e^{-3}$
**C3**	HNF1_Q6	253	Genes with promoter regions [-2kb,2kb] around transcription start site containing the motif WRGTTAATNATTAACNNN which matches annotation for TCF1: transcription factor 1, hepatic; LF-B1, hepatic nuclear factor (HNF1), albumin proximal factor	4 (DMD, RORA, NTF3, HTR7)	0.016	$2.51\ e^{-5}$	$1.31\ e^{-2}$
**C3**	OCT_C	268	Genes with promoter regions [-2kb,2kb] around transcription start site containing motif CTNATTTGCATAY. Motif does not match any known transcription factor	4 (DMD, LPHN1, KIF13A, POLDIP3)	0.015	$3.14\ e^{-5}$	$1.31\ e^{-2}$
**C4**	MODULE_67	230	Genes in the cancer module 67 (Breast and liver cancer).	4 (HDDC2, NTF3, KIF26B, VPS13D)	0.017	$1.73\ e^{-5}$	$1.48\ e^{-2}$
**C4**	MODULE_279	141	Genes in the cancer module 279 (Breast cancer).	3 (DDX18, DMD, KIF13A)	0.021	$1.19\ e^{-4}$	$4.63\ e^{-2}$
**C4**	MODULE_334	166	Genes in the cancer module 334 (Breast cancer).	3 (DDX18, DMD, KIF13A)	0.018	$1.93\ e^{-4}$	$4.63\ e^{-2}$
**C4**	GCM_CSNK1D	32	Neighbourhood of CSNK1D	2 (EVI5, POLDIP3)	0.062	$2.16\ e^{-4}$	$4.63\ e^{-2}$
**C4**	GCM_CSNK1A1	36	Neighbourhood of CSNK1A1	2 (TBC1D15, POLDIP3)	0.055	$2.73\ e^{-4}$	$4.69\ e^{-2}$
**C6**	KRAS.BREAST_UP.V1_DN	145	Genes down-regulated in epithelial breast cancer cell lines over-expressing an oncogenic form of KRAS [Gene ID=3845] gene.	3 (NTF3, FOX5, FGGY)	0.021	$1.3\ e^{-4}$	$2.45\ e^{-2}$
**C6**	PRC2_EED_UP.V1_UP	194	Genes up-regulated in TIG3 cells (fibroblasts) upon knockdown of EED [Gene ID=8726] gene.	3 (EVI5, DMD, SLC30A10)	0.015	$3.05\ e^{-4}$	$2.88\ e^{-2}$

C2: Curated gene sets from online pathway databases, publications in PubMed, and knowledge of domain experts; C3: Motif gene sets based on conserved cis-regulatory motifs from a comparative analysis of the human, mouse, rat, and dog genomes; C4: Computational gene sets defined by mining large

collections of cancer-oriented microarray data; C6: Oncogenic signatures defined directly from microarray gene expression data from cancer gene perturbations (Mootha, 2003; Subramanian, 2005).

http://software.broadinstitute.org/cancer/software/gsea/wiki/index.php/MSigDB_collections

Supplementary Table 9. Summary of resection and asymmetry at intra-chromosomal fusions.

ID PAPER	5p-5p	17p-17p	XpYp-XpYp	TOTAL	5p_TL	17p_TL	XpYp_TL
**CLL1**	1	3	1	5	4.43	2.26	2.42
**CLL2**	1	2	1	4	2.25	1.76	2.29
**CLL3**	0	2	3	5	2.12	1.67	1.34
**CLL4**	0	0	0	0	6.62	6.75	5.59
**CLL5**	1	2	0	3	1.93	1.85	1.04
**CLL6**	1	1	3	5	3.32	3.77	2.34
**CLL7**	0	4	2	6	3.4	2.66	3.05
**CLL8**	0	1	0	1	3.92	2.9	2.96
**CLL9**	3	0	0	3	2.25	1.81	1.74
**TOTAL intra-chromosomal events**	7	15	10	32			
**TOTAL fusion junctions**	14	30	20	64			
**Mean resection**	2442	1917	1047				
**Mean asymmetry**	1408	1240	694.7				
**One sample t-test Difference to 0 of asymmetry P value (two tailed)**	0.0003	0.0004	0.0095				

Supplementary Table 10. Resection and asymmetry at intra-chromosomal fusions

TELOMERE	PATIENT	Resection FJ_1	Resection FJ_2	Distance CpG FJ_1	Distance CpG FJ_2	Asymmetry FJ_1-2
**17p**	CLL1	2083	1194			889
**17p**	CLL1	1865	1884			19
**17p**	CLL1	1194	2083			889
**17p**	CLL2	788	1548			760
**17p**	CLL2	224	2952			2728
**17p**	CLL3	2811	2695			116
**17p**	CLL3	2099	2211			112
**17p**	CLL5	2660	0			2660
**17p**	CLL5	2736	9			2727
**17p**	CLL6	2262	1438			824
**17p**	CLL7	3021	1371			1650
**17p**	CLL7	3022	1220			1802
**17p**	CLL7	3012	2410			602
**17p**	CLL7	224	2952			2728
**17p**	CLL8	2818	2725			93
**5P**	CLL1	2085	2922	797	1634	837
**5P**	CLL2	3338	1565	2050	277	1773
**5P**	CLL5	2590	1873	1302	585	717
**5P**	CLL6	1644	3266	356	1978	1622
**5P**	CLL9	3327	1382	2039	94	1945
**5p**	CLL9	2087	3271	799	1983	1184
**5p**	CLL9	1530	3308	242	2020	1778
**XpYp**	CLL1	61348	60260			1088
**XpYp**	CLL2	60959	60838			121
**XpYp**	CLL3	61633	61357			276

**XpYp**	CLL3	61634	60160			1474
**XpYp**	CLL3	61646	61247			399
**XpYp**	CLL6	61630	60034			1592
**XpYp**	CLL6	61649	61513			136
**XpYp**	CLL6	61672	Xp tel			1634
**XpYp**	CLL7	60621	60671			50
**XpYp**	CLL7	61302	61479			177

Supplementary Table 11. Microhomology at the fusion junction.

**Type of event**	**00**	**0**	**1**	**1/2T**	**2T**	**2G**	**2A**
**Number of values**	12	303	32	25	315	43	9
**Mean**	0.83	1.64	4.09	3.56	1.47	9.12	2.78
**Std. Deviation**	0.83	1.56	3.22	2.66	1.44	5.61	2.49
**Std. Error**	0.24	0.09	0.57	0.53	0.08	0.86	0.83
**Type of event**	00	0	1	1/2	2T	2G	2A
**00: Tel-Tel**	x	ns	**	**	ns	***	ns
**0: Subtel-Tel**	x	x	***	**	ns	***	ns
**1: Intra**	x	x	x	ns	***	ns	ns
**1/2T: Intra/Inter**	x	x	x	x	***	ns	ns
**2T: Telomeric**	x	x	x	x	x	***	*
**2G: Genomic**	x	x	x	x	x	x	ns
**2A: Ancestral**	x	x	x	x	x	x	x
**Not all groups passed normality test. Non-parametric ANOVA Kruskal-Wallis *p*<0.001 and Dunn's Multiple Comparison Test.**							

Supplementary References

1. Oscier D, Wade R, Davis Z, Morilla A, Best G, Richards S, et al. Prognostic factors identified three risk groups in the LRF CLL4 trial, independent of treatment allocation. Haematologica. 2010;95(10):1705-12.
2. Howard DR, Munir T, McParland L, Rawstron AC, Milligan D, Schuh A, et al. Results of the randomized phase IIB ARCTIC trial of low-dose rituximab in previously untreated CLL. Leukemia. 2017.
3. Munir T, Howard DR, McParland L, Pocock C, Rawstron AC, Hockaday A, et al. Results of the randomized phase IIB ADMIRE trial of FCR with or without mitoxantrone in previously untreated CLL. Leukemia. 2017.
4. Capper R, Britt-Compton B, Tankimanova M, Rowson J, Letsolo B, Man S, et al. The nature of telomere fusion and a definition of the critical telomere length in human cells. Genes & development. 2007;21(19):2495-508.
5. Lin TT, Letsolo BT, Jones RE, Rowson J, Pratt G, Hewamana S, et al. Telomere dysfunction and fusion during the progression of chronic lymphocytic leukemia: evidence for a telomere crisis. Blood. 2010;116(11):1899-907.
6. Letsolo BT, Rowson J, Baird DM. Fusion of short telomeres in human cells is characterized by extensive deletion and microhomology, and can result in complex rearrangements. Nucleic acids research. 2010;38(6):1841-52.
7. Liddiard K, Ruis B, Takasugi T, Harvey A, Ashelford KE, Hendrickson EA, et al. Sister chromatid telomere fusions, but not NHEJ-mediated inter-chromosomal telomere fusions, occur independently of DNA ligases 3 and 4. Genome Res. 2016;26(5):588-600.
8. Robinson JT, Thorvaldsdottir H, Winckler W, Guttman M, Lander ES, Getz G, et al. Integrative genomics viewer. Nat Biotechnol. 2011;29(1):24-6.
9. Subramanian A, Tamayo P, Mootha VK, Mukherjee S, Ebert BL, Gillette MA, et al. Gene set enrichment analysis: a knowledge-based approach for interpreting genome-wide expression profiles. Proc Natl Acad Sci U S A. 2005;102(43):15545-50.
10. Cibulskis K, Lawrence MS, Carter SL, Sivachenko A, Jaffe D, Sougnez C, et al. Sensitive detection of somatic point mutations in impure and heterogeneous cancer samples. Nat Biotechnol. 2013;31(3):213-9.
11. Larson DE, Harris CC, Chen K, Koboldt DC, Abbott TE, Dooling DJ, et al. SomaticSniper: identification of somatic point mutations in whole genome sequencing data. Bioinformatics. 2012;28(3):311-7.
12. Klambauer G, Schwarzbauer K, Mayr A, Clevert DA, Mitterecker A, Bodenhofer U, et al. cn.MOPS: mixture of Poissons for discovering copy number variations in next-generation sequencing data with a low false discovery rate. Nucleic Acids Res. 2012;40(9):e69.
13. Pepper C, Ward R, Lin TT, Brennan P, Starczynski J, Musson M, et al. Highly purified CD38+ and CD38- sub-clones derived from the same chronic lymphocytic leukemia patient have distinct gene expression signatures despite their monoclonal origin. Leukemia. 2007;21(4):687-96.
14. Durig J, Naschar M, Schmucker U, Renzing-Kohler K, Holter T, Huttmann A, et al. CD38 expression is an important prognostic marker in chronic lymphocytic leukaemia. Leukemia. 2002;16(1):30-5.
15. Kern W, Bacher U, Haferlach C, Alpermann T, Dicker F, Schnittger S, et al. Frequency and prognostic impact of the aberrant CD8 expression in 5,523 patients with chronic lymphocytic leukemia. Cytometry B Clin Cytom. 2012;82(3):145-50.
16. Dzhagalov I, Giguère V, He Y-W. Lymphocyte Development and Function in the Absence of Retinoic Acid-Related Orphan Receptor α. The Journal of Immunology. 2004;173(5):2952-9.
17. Baskar S, Kwong KY, Hofer T, Levy JM, Kennedy MG, Lee E, et al. Unique cell surface expression of receptor tyrosine kinase ROR1 in human B-cell chronic lymphocytic leukemia. Clinical

cancer research : an official journal of the American Association for Cancer Research. 2008;14(2):396-404.
18. Daneshmanesh AH, Mikaelsson E, Jeddi-Tehrani M, Bayat AA, Ghods R, Ostadkarampour M, et al. Ror1, a cell surface receptor tyrosine kinase is expressed in chronic lymphocytic leukemia and may serve as a putative target for therapy. Int J Cancer. 2008;123(5):1190-5.
19. Daneshmanesh AH, Hojjat-Farsangi M, Moshfegh A, Khan AS, Mikaelsson E, Osterborg A, et al. The PI3K/AKT/mTOR pathway is involved in direct apoptosis of CLL cells induced by ROR1 monoclonal antibodies. British journal of haematology. 2015;169(3):455-8.
20. Zhu Y, McAvoy S, Kuhn R, Smith DI. RORA, a large common fragile site gene, is involved in cellular stress response. Oncogene. 2006;25(20):2901-8.
21. Matsuzaki H, Fujimoto T, Ota T, Ogawa M, Tsunoda T, Doi K, et al. Tespa1 is a novel inositol 1,4,5-trisphosphate receptor binding protein in T and B lymphocytes. FEBS Open Bio. 2012;2:255-9.
22. Bilban M, Heintel D, Scharl T, Woelfel T, Auer MM, Porpaczy E, et al. Deregulated expression of fat and muscle genes in B-cell chronic lymphocytic leukemia with high lipoprotein lipase expression. Leukemia. 2006;20(6):1080-8.
23. Nikitin EA, Malakho SG, Biderman BV, Baranova AV, Lorie YY, Shevelev AY, et al. Expression level of lipoprotein lipase and dystrophin genes predict survival in B-cell chronic lymphocytic leukemia. Leuk Lymphoma. 2007;48(5):912-22.
24. Kamiguti AS, Serrander L, Lin K, Harris RJ, Cawley JC, Allsup DJ, et al. Expression and Activity of NOX5 in the Circulating Malignant B Cells of Hairy Cell Leukemia. The Journal of Immunology. 2005;175(12):8424-30.
25. Roy K, Wu Y, Meitzler JL, Juhasz A, Liu H, Jiang G, et al. NADPH oxidases and cancer. Clin Sci (Lond). 2015;128(12):863-75.
26. Liu Y, Wang R, Zhang L, Li J, Lou K, Shi B. The lipid metabolism gene FTO influences breast cancer cell energy metabolism via the PI3K/AKT signaling pathway. Oncol Lett. 2017;13(6):4685-90.
27. Rozovski U, Hazan-Halevy I, Barzilai M, Keating MJ, Estrov Z. Metabolism pathways in chronic lymphocytic leukemia. Leuk Lymphoma. 2016;57(4):758-65.
28. Jitschin R, Hofmann AD, Bruns H, Giessl A, Bricks J, Berger J, et al. Mitochondrial metabolism contributes to oxidative stress and reveals therapeutic targets in chronic lymphocytic leukemia. Blood. 2014;123(17):2663-72.
29. Edling AE, Nanavati T, Johnson JM, Tuohy VK. Human and murine lymphocyte neurotrophin expression is confined to B cells. J Neurosci Res. 2004;77(5):709-17.
30. Jacob B, Osato M, Yamashita N, Wang CQ, Taniuchi I, Littman DR, et al. Stem cell exhaustion due to Runx1 deficiency is prevented by Evi5 activation in leukemogenesis. Blood. 2010;115(8):1610-20.
31. Baron BW, Anastasi J, Bies J, Reddy PL, Joseph L, Thirman MJ, et al. GFI1B, EVI5, MYB--additional genes that cooperate with the human BCL6 gene to promote the development of lymphomas. Blood Cells Mol Dis. 2014;52(1):68-75.
32. Hofman IJF, van Duin M, De Bruyne E, Fancello L, Mulligan G, Geerdens E, et al. RPL5 on 1p22.1 is recurrently deleted in multiple myeloma and its expression is linked to bortezomib response. Leukemia. 2017;31(8):1706-14.
